# Association between ultrasound-detected synovitis and knee pain: a population-based case–control study with both cross-sectional and follow-up data

**DOI:** 10.1186/s13075-017-1486-7

**Published:** 2017-12-19

**Authors:** Aliya Sarmanova, Michelle Hall, Gwen S. Fernandes, Archan Bhattacharya, Ana M. Valdes, David A. Walsh, Michael Doherty, Weiya Zhang

**Affiliations:** 1 Division of Rheumatology, Orthopaedics and Dermatology, School of Medicine, University of Nottingham, Nottingham, UK; 2 Arthritis Research UK Pain Centre, Nottingham, UK; 3 Arthritis Research UK Centre for Sports, Exercise and Osteoarthritis, Nottingham, UK; 4 School of Health Sciences, University of Nottingham, Nottingham, UK; 5 NIHR Nottingham Biomedical Research Centre, Nottingham, UK

**Keywords:** Knee pain, Synovial changes, Synovitis, Ultrasound, Osteoarthritis, Cohort study

## Abstract

**Background:**

An important role for synovial pathology in the initiation and progression of knee osteoarthritis has been emphasised recently. This study aimed to examine whether ultrasonography-detected synovial changes associate with knee pain (KP) in a community population.

**Methods:**

A case–control study was conducted to compare people with early KP (*n* = 298), established KP (*n* = 100) or no KP (*n* = 94) at baseline. Multinomial logistic regression was used to estimate the odds ratio (OR) and 95% confidence interval (CI) between groups adjusted for radiographic osteoarthritis (ROA) severity and other confounding factors. After 1 year, 255 participants with early and established KP completed the follow-up questionnaire for changes in KP. Logistic regression with adjustment was used to determine predictors of KP worsening.

**Results:**

At baseline, effusion was associated with early KP (OR 2.64, 95% CI 1.57–4.45) and established KP (OR 5.07, 95% CI 2.74–9.38). Synovial hypertrophy was also associated with early KP (OR 5.43, 95% CI 2.12–13.92) and established KP (OR 13.27, 95% CI 4.97–35.43). The association with effusion diminished when adjusted for ROA. Power Doppler signal was uncommon (early KP 3%, established KP 2%, controls 0%). Baseline effusion predicted worsening of KP at 1 year (OR 1.95, 95% CI 1.05–3.64). However, after adjusting for ROA, the prediction was insignificant (adjusted OR 0.95, 95% CI 0.44–2.02).

**Conclusions:**

Ultrasound effusion and synovial hypertrophy are associated with KP, but only effusion predicts KP worsening. However, the association/prediction is not independent from ROA. Power Doppler signal is uncommon in people with KP. Further study is needed to understand whether synovitis is directly involved in different types of KP.

**Electronic supplementary material:**

The online version of this article (doi:10.1186/s13075-017-1486-7) contains supplementary material, which is available to authorized users.

## Background

Knee pain (KP) affects one in four people aged over 55 years, of whom 10% have mild-to moderate disability [1]. KP is the main symptom of knee osteoarthritis (OA), and the prevalence of OA continues to rise because of increasing longevity and obesity, causing a significant socio-economic burden [2, 3]. Individuals with KP but normal radiographs are more likely to develop radiographic OA (ROA) later, suggesting that KP can be a symptom prior to the observable structure damage of OA, not necessarily a consequence of structural alteration sufficient to show on an X-ray image [4, 5]. The classification criteria for OA probably exclude people with early or structurally mild disease, which might also contribute to KP in the community [1].

An important role for synovial pathology, specifically synovitis, in the initiation and progression of knee OA has been emphasised recently [6–9]. Ultrasonography (US) is a commonly used imaging modality to detect soft-tissue changes in the knee [10]. It is relatively inexpensive, involves a short examination time, and correlates in people with knee OA with histological findings [11, 12] and magnetic resonance imaging (MRI) [13, 14]. With the increasing focus on precision medicine, synovial pathology has been proposed as a target for intervention and as a biomarker for people who require anti-inflammatory therapy for OA [15]. Therefore, it is important to know whether US-detected synovial changes (USSCs) associate with KP and predict changes in symptoms over time in people who might benefit from targeted treatments [16]. However, evidence for this association in established OA is conflicting [17–19]. Moreover, previous studies have reported that radiographic structural changes, a strong risk factor for KP [20, 21], also associate with USSCs [22]. Therefore, in order to explore the relationships between KP and synovial changes it is important to account for ROA and other peripheral risk factors such as muscle strength. While muscle weakness associates with knee OA [23], this association is independent of ROA severity [24, 25]. Also, USSCs may differ in early and advanced OA [26, 27], and the lack of studies in people recruited from the community [28] may influence the generalisability of previous results [29]. Furthermore, whether USSCs predict changes in KP has not been examined [30]. The current study aimed to examine whether community-derived people with early or established KP are more likely to have USSCs, specifically effusion, synovial hypertrophy and Power Doppler (PD) signal, compared to controls without KP and to explore whether USSCs predict/associate with subsequent KP worsening.

## Methods

This study was approved by the Nottingham University Hospitals NHS Trust and Nottingham Research Ethics Committee 1 (Ref 14/EM/0015) and was registered on ClinicalTrials.gov (NCT02098070) [31].

### Study design and participant selection

Participants for this case–control study were selected from the Knee Pain and Related Health in the Community (KPIC) Study, an ongoing prospective cohort study that included 9506 men and women aged ≥ 40 years at baseline. A second questionnaire was posted 1 year later to the 6716 participants who indicated willingness to receive a further questionnaire and who were alive.

Participants were selected according to current KP status irrespective of subsequent radiographic findings. “Early KP” was defined as pain commencing within the past 3 years regardless of pain severity. “Established KP” was moderate to severe KP of more than 3 years in duration. KP-free controls reported “no KP” in the past 5 years. Exclusions were: known terminal illness; severe psychiatric illness or dementia; knee arthroplasty; major prior knee/lower limb injury; or current pregnancy.

Selection for the early KP group was from all participants who met the inclusion criteria and agreed to participate in clinical assessments. Participants for the established KP and no KP groups were frequency matched to early KP participants by age and gender. Random selection was undertaken if more than one participant was eligible for matching. In addition, all participants who reported incident KP at 1 year and met the inclusion criteria were invited for assessment and included in the early KP group.

Age, gender, height, pain status and use of prescribed and/or over-the-counter analgesics (e.g. paracetamol, NSAIDs and COX-2 selective inhibitors, opioids) were self-reported in the postal questionnaire. At baseline all participants had US, radiographic and muscle strength assessments.

### Pain assessment

KP was defined as pain in or around a knee on most days for at least a month [32, 33]. A 0–10 numerical rating scale (NRS) was used to assess pain intensity in the past month.

A patient global assessment (PGA) of KP change at year 1 was defined by response to the question: “Since it has started, do you think the severity of your knee pain has overall … greatly improved/slightly improved/remained the same/worsened”.

The index knee was the only or most painful knee. For equal bilateral KP or no KP participants, the index knee was selected randomly. Data on USSCs, ROA and muscle strength were presented for index knees only.

### Ultrasound assessment

US examination was performed by two assessors (MH, AS) using the Toshiba Aplio SSA-770A machine with a multi-frequency (7–12 MHz) linear array transducer. The same equipment and software were used during the whole study.

The assessment was performed with knee flexion of approximately 20–30° and included the supra-patellar recess and medial and lateral tibio-femoral spaces. USSCs were defined using OMERACT-7 definitions (Additional file 1) [34]. The depth of synovial thickness (hypertrophy) and effusion were measured on a continuous scale at their maximal diameter in millimetres using the longitudinal axis. Absolute values were dichotomised as absent (< 4 mm) or present (≥ 4 mm) according to EULAR recommendations [35]. PD assessment was focused on areas of synovial hypertrophy and recorded as absent or present. Only one value per joint was recorded for each US feature (maximum value across three areas scanned). It has been reported previously that overall agreement between synovial hypertrophy detected in these three areas of the knee and synovitis detected using arthroscopy (“gold standard”) was 97% with a non-significant difference in sensitivity between the three compartments [36].

### Radiographic OA assessment

Bilateral weight-bearing semi-flexed posterior–anterior tibio-femoral views using a Rosenberg template and 30° flexion skyline patello-femoral views were undertaken using standardised protocols. The Nottingham logically derived line drawing atlas (LDLDA) [37, 38] was used to score joint space narrowing (JSN) in medial and lateral tibio-femoral and medial and lateral patello-femoral articulations (each scored –1 to 5) and osteophytes (at eight sites in the three compartments, each scored 0–5). The scores for all three compartments, ignoring –1 values for JSN (i.e. joint space widening), were summated as a global score for each knee. Presence of ROA was defined as definite JSN (grade 2) plus definite osteophyte (grade 2) in any compartment (tibio-femoral or patello-femoral).

### Muscle strength assessment

Maximal isometric strength of quadriceps and hip abductor muscles was tested using a Nicholas Manual Muscle Tester (MMT) (Lafayette Instruments) three times on each leg and then the mean values were calculated for each side [39]. Normal tertiles of the quadriceps and hip abductor strength were calculated from the pain-free controls separately for men and women.

All assessments were independent, standardised and blinded to participants’ characteristics including pain status. USSCs, radiographic score and muscle strength in index knees only were used for analysis.

### Statistical analysis

#### Sample size

##### Baseline cross-sectional study

An unbalanced (2:1:1 for “early KP”, “established KP”, “no KP”) one-way ANOVA design was applied to ensure sufficient early KP cases for the cohort study. The effect sizes reported by Hall et al. [22] were used to calculate the sample size (i.e. mean (SD) was 1.0 (1.9), 6.7 (3.3) and 0.7 (1.5) for synovial hypertrophy in the three groups respectively). Considering 90% power with 5% type I error, 80 participants were required for the primary analysis to detect the minimum difference between the three groups (40:20:20).

##### One-year follow-up study

For the risk prediction model, the sample size was calculated based on the logistic model with one predictor adjusted with three covariates (e.g. age, gender and BMI) assuming that there is a correlation between covariates (*r* = 0.3). The study was powered for an odds ratio (OR) as small as 1.7 for synovial hypertrophy assuming that the probability of worsening of KP is 14%,[28] (Ingham SL, Zhang W, Doherty M: Natural history of knee pain in the Nottingham community: health states and transition probabilities in a 10 year retrospective cohort study. Unpublished manuscript, available on request). With 80% power and less than 5% type I errors, 211 participants are required.

#### Primary analysis

##### Baseline cross-sectional study

The association between US features and KP was estimated using the OR and 95% confidence interval (CI). Multi-nominal logistic regression was used to estimate ORs between the early, established and no KP groups with the no KP group as reference. The OR was adjusted for age, gender, BMI, global X-ray score and quadriceps strength.

##### One-year follow-up study

Potential baseline predictors for KP worsening as defined by PGA were examined using multivariate logistic regression analysis with adjustment for age, gender and BMI. Sensitivity analysis was undertaken using an alternative definition of KP worsening, defined by any increase in KP from baseline on a NRS.

#### Other analyses

The cross-sectional association of USSCs with radiographic severity was examined using a two-level generalised linear mixed model to adjust for cluster effects (i.e. the difference between the three groups).

#### Reliability

The unweighted kappa statistic was used for dichotomous data and concordance correlation for continuous data [40, 41]. For the inter-observer reliability test, two assessors (MH, AS) blindly, independently and consecutively carried out the grey-scale and PD US examination on the same day (16 individuals, 32 knees). Intra-observer reliability (AS) was examined by scanning four volunteers (eight knees) on two separate days within a 7-day period. Inter-observer and intra-observer agreement for radiographic scoring (AS, GSF) was examined using images from 21 participants with different radiographic severity (40 knees). Muscle strength reliability testing was performed on 10 volunteers.

All statistical analyses were undertaken using SAS software v9.4 licensed to the University of Nottingham.

## Results

### Baseline cross-sectional study

#### Demographics

At baseline 495 participants were recruited, of whom 298 had early KP, 103 established KP and 94 no KP. Of those with early KP, 219 were recruited at baseline and 79 were incident cases identified during follow-up. Age and gender were distributed equally among the three groups. However, a graded increase from no KP to early KP and then to established KP groups was observed for BMI, pain severity, ROA, low quadriceps and hip abductor strength, and use of analgesics (Table 1).

**Table 1:** Characteristics of the study population

	No knee pain	Early knee pain	Established knee pain	*p* value
*N*	94	298	103	
Age (years), mean (SD)	60.98 (9.81)	61.42 (9.66)	59.53 (10.04)	0.2992*
Women, *n* (%)	58 (61.70)	179 (60.07)	63 (61.17)	0.9509**
BMI (kg/m^2^), mean (SD)	26.78 (4.49)	28.85 (5.70)	31.96 (6.49)	< 0.0001*
Pain severity (NRS 0–10)^a^, mean (SD)		4.55 (2.52)	7.40 (2.14)	< 0.0001^†^
Global X-ray score (mm, 0–60), mean (SD)	2.24 (3.08)	5.72 (7.00)	11.28 (9.26)	< 0.0001*
Radiographic OA, *n* (%)	7 (7.45)	80 (26.85)	49 (47.57)	< 0.0001**
Muscle strength^b^, *n* (%)				
Quadriceps strength (kg, lowest tertile)	33 (35.11)	99 (33.22)	65 (63.11)	< 0.0001**
Hip abductor strength (kg, lowest tertile)	33 (35.11)	119 (39.93)	68 (66.02)	< 0.0001**
Use of analgesics, *n* (%)				
Prescribed NSAIDs	3 (3.19)	19 (6.38)	15 (14.56)	0.0018
Opioids	3 (3.19)	44 (14.77)	22 (21.36)	0.0005
Over-the-counter NSAIDs	12 (12.77)	68 (22.82)	32 (31.07)	0.0021

Groups matched by age and gender. *p* values adjusted for multiple testing using the bootstrap method

*SD* standard deviation, *NRS* numerical rating scale, *BMI* body mass index, *OA* osteoarthritis, *NSAID* non-steroidal anti-inflammatory drug

^†^ *t* test

*Test for linear trend

**Cochran–Armitage test for trend

^a^Average pain severity in the past month

^b^Lowest tertile values for muscle strength tests: quadriceps strength < 17.6 kg for men and < 10.7 kg for women; hip abductor strength < 12.8 kg and < 8.2 kg, respectively

#### Reliability

The level of inter-observer agreement was moderate for effusion and substantial for synovial hypertrophy (κ = 0.44 and 0.61, respectively). Intra-observer agreement for effusion was moderate (κ = 0.50). There were insufficient data to calculate kappa statistics for synovial hypertrophy (mean difference between measurements 0.3 mm (SD 0.7)) and PD signal. Inter-observer and intra-observer agreement on radiographic scoring was substantial (all κ ≥ 0.78). Both inter-rater and intra-rater reliability in muscle strength testing ranged from adequate to excellent (0.64–0.94).

#### Ultrasound synovial features and KP

Effusion ≥ 4 mm was associated with KP, but this association diminished after adjustment for age, gender, BMI, ROA severity and quadriceps strength (Table 2). Synovial hypertrophy also associated with KP and this association remained statistically significant after adjustment for age, gender, BMI and radiographic severity. Adjusted ORs (95% CIs) were 3.17 (1.17-8.53) for early KP and 4.97 (1.66-14.86) for established KP. There was a strong association between ROA and KP. ORs adjusted for age, gender and BMI were 4.37 (95% CI 1.89–10.13) and 11.82 (95% CI 4.71–29.66) for early and established KP respectively. There were no interactions between effusion/hypertrophy and radiographic severity (all *p* > 0.05, data not presented). Additional adjustment for low quadriceps strength and analgesic use did not change the strength of association (Additional file 2).

**Table 2:** Ultrasound synovial features at baseline and associations with knee pain

	No knee pain	Early knee pain	Established knee pain	*p* for trend
Effusion				
Mean ± SD (mm)	3.02 (2.10)	4.48 (3.64)	5.89 (3.48)	< 0.0001
≥ 4 mm, *n* (%)	23 (24.47)	136 (45.64)	64 (62.14)	< 0.0001
≥ 4 mm, OR (95% CI)	1	2.64 (1.57–4.45)	5.07 (2.74–9.38)	
≥ 4 mm, aOR (95% CI)	1	1.90 (1.07–3.39)	1.92 (0.92–4.00)	
Synovial hypertrophy				
Mean ± SD (mm)	0.65 (1.56)	2.01 (2.66)	3.57 (3.49)	< 0.0001
≥ 4 mm, *n* (%)	5 (5.32)	69 (23.15)	44 (42.72)	< 0.0001
≥ 4 mm, OR (95% CI)	1	5.43 (2.12–13.92)	13.27 (4.97–35.43)	
≥ 4 mm, aOR (95% CI)	1	3.17 (1.17–8.53)	4.97 (1.66–14.86)	
Power Doppler signals, *n* (%)	0	10 (3.36)	2 (1.94)	0.4252

*aOR* odds ratios adjusted for age, gender, BMI, quadriceps strength and radiographic osteoarthritis scores, *CI* confidence interval, *OR* odds ratio, *SD* standard deviation

### One year follow-up study

After 1 year, 181 (83%) participants with early KP and 74 (76%) participants with established KP completed the follow-up questionnaire. There was no difference between those who returned the questionnaire and the entire population (Additional file 3). After 1 year, 18% of people with early KP reported that their pain had worsened (*n* = 32 out of 181) and 42% of people with established KP reported worsening of pain (*n* = 31 out of 74).

After adjustment for age, gender and BMI, effusion (aOR 1.95, 95% CI 1.05–3.64) and ROA (aOR 4.73 95% CI 2.46 to 9.10) predicted worsening of KP (Table 3). However, the association between effusion and worsening of KP adjustment for analgesic use did not change the strength of association (data not presented).

**Table 3:** Association between baseline risk factors and worsening of knee pain

	Descriptive		OR (95% CI)	
	Stable/improved	Worsened	Crude	Age, gender, BMI-adjusted
*N*	192	63		
Effusion				
Mean (SD) (mm)	4.24 (3.44)	6.20 (4.09)	**1.15 (1.06–1.24)**	**1.11 (1.02–1.20)**
Effusion ≥ 4 mm, *n* (%)	79 (41.58)	40 (63.49)	**2.44 (1.36–4.40)**	**1.95 (1.05–3.64)**
Synovial hypertrophy				
Mean (SD) (mm)	2.06 (2.89)	3.35 (3.35)	**1.14 (1.04–1.24)**	1.09 (0.99–1.20)
Thickness ≥ 4 mm, *n* (%)	45 (23.68)	24 (38.10)	**1.98 (1.08–3.64)**	1.40 (0.72–2.74)
Power Doppler signal, *n* (%)	8 (4.17)	1 (1.59)	0.37 (0.05–3.03)	0.47 (0.06–4.02)
Global radiographic score (0–-60), mean (SD)	5.81 (7.19)	13.58 (9.40)	**1.11 (1.07–1.15)**	**1.10 (1.06–1.14)**
Radiographic OA, *n* (%)	44 (23.16)	39 (62.90)	**5.63 (3.04–10.41)**	**4.73 (2.46–9.10)**
Quadriceps strength (kg, lowest tertile)	66 (34.74)	34 (53.97)	**2.20 (1.24–3.93)**	**1.89 (1.04–3.46)**

Significant associations are highlighted in bold

*BMI* body mass index, *CI* confidence interval, *OA* osteoarthritis, *OR* odds ratio, *SD* standard deviation

The sensitivity analysis using any increase from baseline in NRS for KP also showed that no US feature predicted increased KP (Additional file 4).

### Other results

#### Ultrasound features and radiographic changes

**Fig. 1:**
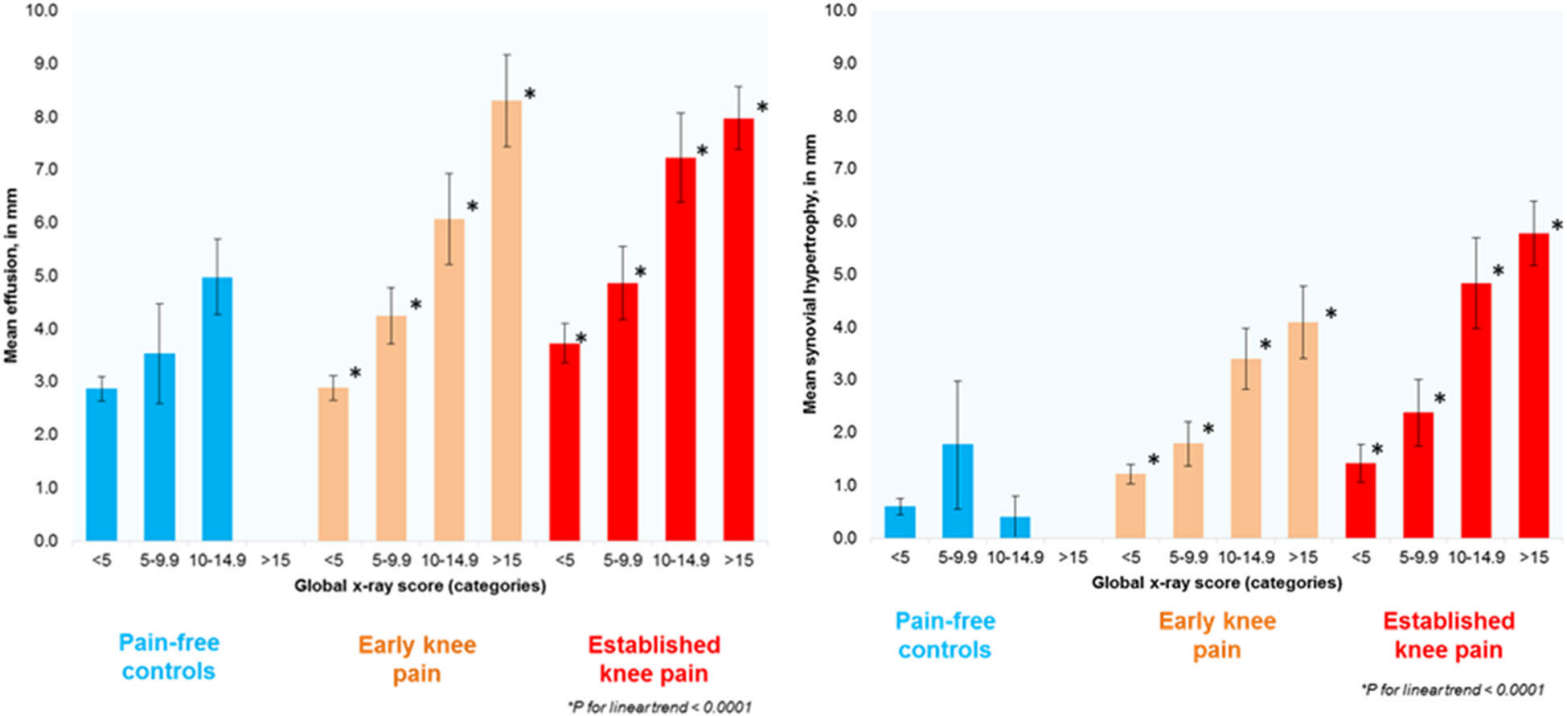
Mean effusion (left) and mean synovial hypertrophy (right) for each group. Note: For global X-ray score (horizontal axis), the scale was categorised as < 5, 5–9.99, 10–14.99 and > 15. Vertical error bars indicate standard error of the mean

At baseline, both effusion and synovial hypertrophy showed dose–response relationships with global ROA scores (Fig. 1). After adjusting for all other confounding factors, the regression coefficients were 0.21 (95% CI 0.17–0.25) for effusion and 0.17 (95% CI 0.13–0.20) for synovial hypertrophy (both *p* < 0.0001).

## Discussion

To our knowledge, this is the first community-based study to investigate USSCs and their association with KP, adjusted for radiographic severity. The main findings are: USSCs associate with KP, and the association is confounded by ROA severity; and effusion and ROA at baseline predict KP worsening at 1 year, but the prediction becomes insignificant after adjusting for ROA.

The association between synovial changes and KP in OA have been investigated previously. In our recent meta-analysis [42], seven out of 10 studies reported a positive association between KP and effusion and two out of six studies reported an association with synovial hypertrophy. However, most studies did not adjust for ROA. Although the prevalence of US-detected synovial pathology (effusion, hypertrophy, PD) showed wide variability between studies, the pooled prevalence of these features was significantly higher in people with knee OA than in people with KP (*p* < 0.05). This prompted the current study to investigate the relationship between US features of “synovitis”, ROA and KP.

Our findings suggest that the association between USSCs (“synovitis”) and KP may be confounded by radiographic structural changes of OA. This is supported by: the strong dose–response association between ROA and synovial hypertrophy; the diminishing association between KP and effusion after adjustment for ROA; and the lack of prediction of USSCs for pain worsening in contrast to the prediction of baseline ROA change for pain worsening over 1 year. This suggests that “synovitis” detected by US is not the main cause of KP but a consequence of the overall pathology of OA that involves all joint tissues. This has been confirmed by the graded ORs from no KP, early KP and established KP (Table 2). Our conclusion is that both US “synovitis” and ROA are risk factors for KP and strongly relate to each other. The positive association between synovial changes and structural severity accords with the MRI findings [43, 44]. Further studies that specifically examine the relationship between synovial change and change in other joint tissues are warranted.

In our study we found that presence of ROA is a prognostic factor that predicts worsening of pain over 1 year. The association between OA structural severity and KP has been confirmed in a number of cross-sectional studies [20] whereas evidence for ROA as a predictor of KP progression remained controversial [45].

Recently, there has been considerable interest in inflammation in OA and the possibility that “synovitis” is a marker for an inflammatory phenotype of symptomatic OA [6, 7]. However, in contrast to rheumatoid arthritis (RA) and other arthropathies that are driven by inflammation, the intensity of inflammation in OA is only modest. Early-morning and inactivity stiffness are relatively short in OA [46] and large effusions are atypical and suggest co-existing inflammatory conditions such as crystal synovitis [47–49]. Furthermore, although synovial hyperplasia and effusion may occur in OA, synovial hyperplasia is more focal than generalised, effusions have relatively low cell counts with a preponderance of mononuclear cells, and marginal cortical erosions do not occur [50, 51]. This contrasts with RA where high cell counts (causing turbidity) with a predominance of neutrophils and development of marginal cortical erosions are characteristic. It is possible that effusion in knee OA in part is non-inflammatory, arising from attrition of lymphatics rather than fluid overproduction due to inflammation [52]. Generalised synovial hypertrophy and strongly positive PD signal are US markers of inflammation in RA [53, 54], the PD signal indicating marked hypervascularity. Although we found a positive association between synovial hypertrophy and KP, the prevalence of the PD signal was very low in both KP groups. Our data align with the perspective of OA as an inherent repair process in which all tissues that comprise the synovial joint, including the synovium and capsule, respond to diverse insults (including biomechanical factors) by producing new tissue [50].

There are several caveats to this study. Firstly, it was designed to primarily determine the association between USSCs and KP, so associations with radiographic features should be interpreted with caution. It is possible that the associations between US and ROA with KP might result from other associated factors. Secondly, pain was re-assessed at just two time points and further longer-term follow-ups are warranted. Thirdly, currently there is no accepted standardised protocol for US assessment. Our study included assessment of three areas (supra-patellar pouch, medial and lateral aspects of the knee) with the maximum value of effusion/hypertrophy recorded per knee. Karim et al. [36] reported previously that these three areas have similar sensitivity for detecting of synovitis compared with synovitis detected using arthroscopy (“gold standard”). However, a more detailed protocol with separate scoring per area or using a multi-compartmental summated score might reveal a different association with KP. Fourthly, US and radiographs cannot examine all joint changes in OA (e.g. bone marrow lesions) and use of MRI, although expensive, would have allowed more detailed and comprehensive assessment of joint abnormalities. Lastly, the reliability of US assessment is an important issue to consider. The level of agreement between observers was not perfect but was at least moderate and in line with an OMERACT reliability exercise [55]. In that study the agreement between 11 experienced sonographers was fair for both effusion and synovial hypertrophy (mean κ = 0.38 and 0.29, respectively) and the intra-rater agreement was moderate for both US features (mean κ = 0.56 and 0.49, respectively) [55]. Unfortunately, PD signals were uncommon in the study population (0% in no KP, 3% in early KP and 2% in established KP), which limited the value of this measure.

## Conclusions

In summary, USSCs (synovitis) are associated with KP and the association is confounded by structural OA. Effusion but not synovial hypertrophy at baseline predicts KP worsening at 1 year but the prediction is not independent of ROA. USSCs are related to radiographic severity of OA but the causal relationship between the two has yet to be established.

## Additional files

Additional file 1: Is a figure showing grey-scale US images of effusion and synovial hypertrophy in the supra-patellar pouch and Power Doppler signal in the lateral tibio-femoral space of the knee. (DOCX 975 kb)

Additional file 2: Is a table presenting ultrasound synovial features and radiographic osteoarthritis and associations with knee pain. (DOCX 36 kb)

Additional file 3: Is a table presenting characteristics of the responders to the follow-up questionnaire at 1 year among people with early and established knee pain recruited at baseline. (DOCX 36 kb)

Additional file 4: Is a table presenting the association between baseline risk factors and increase in pain severity (NRS 0–10). (DOCX 37 kb)

## Abbreviations

ANOVA: Analysis of variance
aOR: Adjusted odds ratio
BMI: Body mass index
CI: Confidence interval
COX: Cyclooxygenase
EULAR: European League Against Rheumatism
JSN: Joint space narrowing
KP: Knee pain
KPIC: Knee Pain and Related Health in the Community
LDLDA: Logically derived line-drawing atlas
MRI: Magnetic resonance imaging
MMT: Nicholas Manual Muscle Tester
NRS: Numeric rating scale
NSAID: Non-steroidal anti-inflammatory drug
OA: Osteoarthritis
OR: Odds ratio
PD: Power Doppler
PGA: Patient global assessment
US: Ultrasound
USSC: Ultrasound-detected synovial change
