# Supplementary material for: Association between ultrasound-detected synovitis and knee pain: a population-based case–control study with both cross-sectional and follow-up data
Source: Arthritis Res Ther. 2017 Dec 19;19:281. doi: 10.1186/s13075-017-1486-7 (PMC5738097; doi:10.1186/s13075-017-1486-7)
Supplement: Supplementary file 1 — Is a figure showing grey-scale US images of effusion and synovial hypertrophy in the supra-patellar pouch and Power Doppler signal in the lateral tibio-femoral space of the knee. (DOCX 975 kb) [file 13075_2017_1486_MOESM1_ESM.docx]

**Additional file 1. Grey-scale US image of an effusion and synovial hypertrophy in the supra-patellar pouch, and Power Doppler signal in the lateral tibio-femoral space of the knee.**


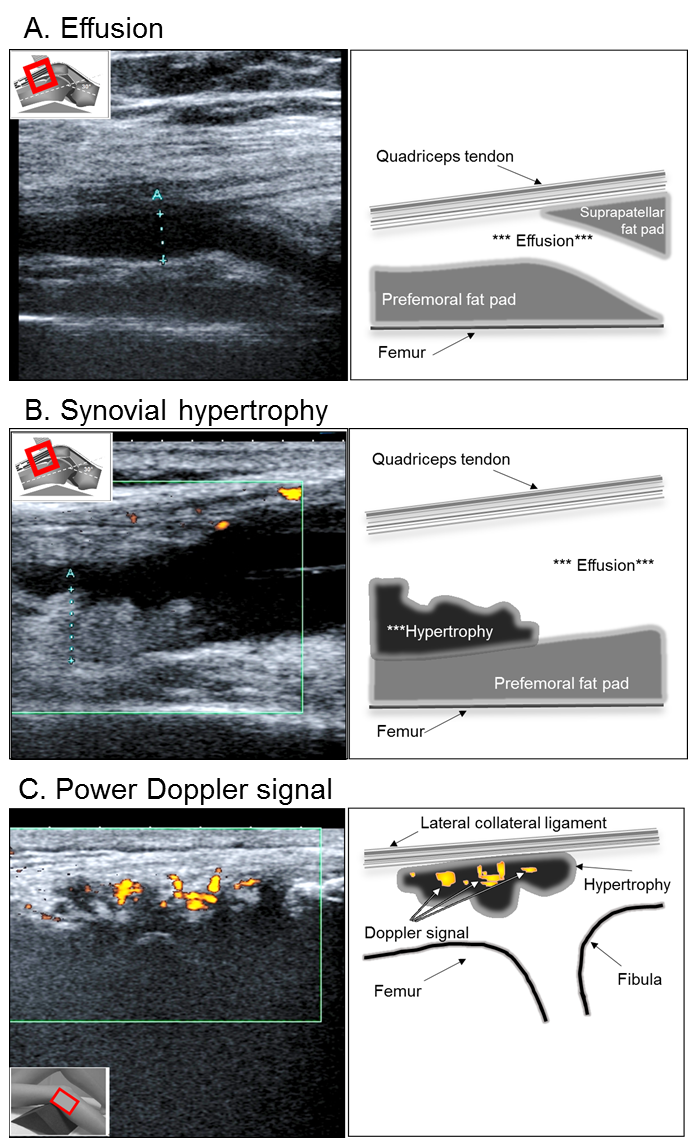


Note: On the left the real US images with detected synovial pathology (taken from study participant); with images of the knee area and position (adapted from ([Bianchi and Martinoli, 2007](#_ENREF_33)), permission granted); on the right are schematic drawings synovial pathology in relation to other joint structures.
