# Supplementary material for: Association between ultrasound-detected synovitis and knee pain: a population-based case–control study with both cross-sectional and follow-up data
Source: Arthritis Res Ther. 2017 Dec 19;19:281. doi: 10.1186/s13075-017-1486-7 (PMC5738097; doi:10.1186/s13075-017-1486-7)
Supplement: Supplementary file 2 — Is a table presenting ultrasound synovial features and radiographic osteoarthritis and associations with knee pain. (DOCX 36 kb) [file 13075_2017_1486_MOESM2_ESM.docx]

**Additional file 2. Ultrasound synovial features and radiographic osteoarthritis and associations with knee pain**

|  | **No Knee Pain** | **Early Knee Pain** | **Established**  **Knee Pain** |
| --- | --- | --- | --- |
| **Effusion** |  |  |  |
| *≥4mm aOR (95%CI)^1^* | 1 | 1.90 (1.07; 3.39) | 1.92 (0.92; 4.00) |
| *≥4mm aOR (95%CI)^2^* | 1 | 1.80 (1.01; 3.22) | 1.77 (0.84; 3.73) |
| **Synovial hypertrophy** |  |  |  |
| *≥4mm aOR (95%CI)^1^* | 1 | 3.18 (1.18; 8.57) | 5.07 (1.70; 15.12) |
| *≥4mm aOR (95%CI)^2^* | 1 | 2.80 (1.03; 7.61) | 4.28 (1.42; 12.90) |
| **ROA** |  |  |  |
| *Crude OR (95%CI)* |  | 4.43 (1.96; 9.98) | 11.02 (4.65; 26.10) |
| *aOR (95%CI)^3^* |  | 4.37 (1.89; 10.13) | 11.82 (4.71; 29.66) |
| *aOR (95%CI)^4^* |  | 2.84 (1.19; 6.80) | 5.77 (2.20; 15.18) |
| *aOR (95%CI)^5^* |  | 2.81 (1.17; 6.75) | 6.15 (2.30; 16.43) |
| *aOR (95%CI)^6^* |  | 2.83 (1.18; 6.83) | 6.11 (2.27; 16.45) |

**Note:** 1 - odds ratios adjusted for age, gender, BMI, quadriceps strength and radiographic OA scores.

2 - odds ratios adjusted for age, gender, BMI, quadriceps strength, radiographic OA scores and analgesic use.

3 - odds ratios adjusted for age, gender and BMI.

4 - odds ratios adjusted for age, gender, BMI and synovial hypertrophy.

5 - odds ratios adjusted for age, gender, BMI, synovial hypertrophy and quadriceps strength.

6 - odds ratios adjusted for age, gender, BMI, synovial hypertrophy, quadriceps strength and analgesic use.
