# Supplementary material for: Association between ultrasound-detected synovitis and knee pain: a population-based case–control study with both cross-sectional and follow-up data
Source: Arthritis Res Ther. 2017 Dec 19;19:281. doi: 10.1186/s13075-017-1486-7 (PMC5738097; doi:10.1186/s13075-017-1486-7)
Supplement: Supplementary file 3 — Is a table presenting characteristics of the responders to the follow-up questionnaire at 1 year among people with early and established knee pain recruited at baseline. (DOCX 36 kb) [file 13075_2017_1486_MOESM3_ESM.docx]

**Additional file 3. Characteristics of the responders to the follow-up questionnaire at one year among people with early and established knee pain recruited at the baseline**

|  | **Total**  **(N=322)** | **Responders**  **(N=255)** |
| --- | --- | --- |
| **Age (years), mean (SD)** | 60.04 (9.63) | 60.94 (9.65) |
| **Women, n (%)** | 197 (61.18) | 156 (61.18) |
| **BMI, mean (SD)** | 29.95 (6.05) | 29.43 (5.61) |
| **Baseline current knee pain severity (NRS 0-10), mean (SD)** | 5.53 (2.67) | 5.30 (2.64) |
| **Radiographic osteoarthritis, n (%)** | 98 (30.43) | 83 (32.94) |

**Note:** * p-value for the difference between responders and the whole source population.

SD - standard deviation; BMI - body mass index; NRS – numerical rating scale (range 0-10).
