# Supplementary material for: Association between ultrasound-detected synovitis and knee pain: a population-based case–control study with both cross-sectional and follow-up data
Source: Arthritis Res Ther. 2017 Dec 19;19:281. doi: 10.1186/s13075-017-1486-7 (PMC5738097; doi:10.1186/s13075-017-1486-7)
Supplement: Supplementary file 4 — Is a table presenting the association between baseline risk factors and increase in pain severity (NRS 0–10). (DOCX 37 kb) [file 13075_2017_1486_MOESM4_ESM.docx]

**Additional file 4. Association between baseline risk factors and increase in pain severity (NRS 0-10)**

|  |  | **Descriptive** | | **ORs (95% CI)** | |
| --- | --- | --- | --- | --- | --- |
|  |  | **Stable/**  **Improved** | **Worsened** | **Crude** | **Age, gender, BMI-adjusted** |
| **N** |  | 193 | 62 |  |  |
| **Effusion** |  |  |  |  |  |
| *Mean in mm (SD)* |  | 4.70 (3.67) | 4.80 (3.83) | 1.01 (0.93; 1.09) | 1.01 (0.92; 1.09) |
| *Effusion≥4mm, n (%)* |  | 88 (45.83) | 31 (50.82) | 1.22 (0.69; 2.17) | 1.15 (0.62; 2.13) |
| **Synovial hypertrophy** |  |  |  |  |  |
| *Mean in mm (SD)* |  | 2.34 (3.04) | 2.52 (3.12) | 1.02 (0.93; 1.12) | 1.02 (0.92; 1.13) |
| *Thickness≥4mm, n (%)* |  | 50 (26.04) | 19 (31.15) | 1.28 (0.68; 2.41) | 1.29 (0.64; 2.60) |
| **Power Doppler Signal, n (%)** |  | 6 (3.11) | 3 (4.84) | 1.59 (0.38; 6.53) | 1.55 (0.36; 6.63) |
| **Global x-ray score (0-60), mean (SD)** |  | 7.55 (8.55) | 8.27 (8.24) | 1.01 (0.98; 1.04) | 1.01 (0.97; 1.05) |
| **Radiographic OA, n (%)** |  | 59 (30.73) | 24 (40.00) | 1.50 (0.82; 2.74) | 1.53 (0.80; 2.94) |

**Note:**  OA – osteoarthritis; SD – standard deviation; BMI – body mass index; ORs –odds ratios; CI –confidence interval.
